# Surgical management of achalasia

**DOI:** 10.1002/ags3.12344

**Published:** 2020-05-25

**Authors:** Kamil Nurczyk, Marco G. Patti

**Affiliations:** ^1^ Department of Surgery University of North Carolina at Chapel Hill Chapel Hill NC USA; ^2^ 2nd Department of General and Gastrointestinal Surgery, and Surgical Oncology of the Alimentary Tract Medical University of Lublin Lublin Poland; ^3^ Department of Medicine University of North Carolina at Chapel Hill Chapel Hill NC USA

**Keywords:** dor fundoplication, esophageal achalasia, heller myotomy, peroral endoscopic myotomy, pneumatic dilatation

## Abstract

Esophageal achalasia is a primary esophageal motility disorder characterized by lack of peristalsis and by incomplete or absent relaxation of the lower esophageal sphincter in response to swallowing. The cause of the disease is unknown. The goal of treatment is to eliminate the functional outflow obstruction at the level of the gastroesophageal junction, therefore allowing emptying of the esophagus into the stomach. They include the laparoscopic Heller myotomy with partial fundoplication, pneumatic dilatation, and peroral endoscopic myotomy. Esophagectomy is considered as a last resort for patients who have failed prior therapeutic attempts. In this evidence and experience‐based review, we will illustrate the technique and results of the surgical treatment of esophageal achalasia and compare it to the other available treatment modalities.

## Graphical abstract

Esophageal achalasia is a primary esophageal motility disorder characterized by lack of peristalsis and by incomplete or absent relaxation of the lower esophageal sphincter in response to swallowing. Available treatment methods include the laparoscopic Heller myotomy with partial fundoplication, pneumatic dilatation, peroral endoscopic myotomy, and esophagectomy. In this evidence‐ and experience‐based review, we will illustrate the technique and results of the surgical treatment of esophageal achalasia, and compare it to the other available treatment modalities.

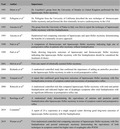

## 1 INTRODUCTION

Esophageal achalasia is a rare disease that affects approximately one in 100 000 people, regardless of gender or race. However, in some geographical locations such as Brazil, it is much more common in connection to the high prevalence of Chagas disease.^1^ The incidence of achalasia increases with patient age.^2^ In addition, it seems that the prevalence of this disease is increasing, probably due to improvements in diagnostic modalities.^3^

In normal conditions, the lower esophageal sphincter (LES) relaxes in response to swallowing. This physiological mechanism is dependent on neurogenic control of the esophagus and LES through the myenteric plexus, combining excitatory acetylcholine neurons, inhibitor nitric oxide, and VIP neurons. Idiopathic achalasia is due to the degeneration of inhibitory neurons, which are involved in the relaxation of LES. As a result, the LES does not relax properly in response to swallowing, and it is often hypertensive. In addition, there is a lack of esophageal peristalsis.

The lack of peristalsis and the impaired LES relaxation impair the transit of the food bolus from the esophagus into the stomach, leading eventually to dilatation of the esophageal body. Almost every patient experiences dysphagia, which often leads to weight loss. Regurgitation of undigested food is also a common ailment, and may cause complications such as hoarseness, coughing, wheezing, and pneumonia. Up to 50% of patients with achalasia also experience heartburn, which is caused by fermentation of retained food in the esophagus. Chest discomfort or pain can also occur, and they are caused by esophageal distension, which usually increases while eating.^4^ The severity of achalasia symptoms is evaluated using the Eckardt score.

## 2 PREOPERATIVE WORK‐UP

A comprehensive evaluation of every patient should be carried out to confirm the initial diagnosis suggested by the symptoms, and should include: esophagogastroduodenoscopy (EGD), barium swallow, esophageal manometry, and in some cases ambulatory 24‐hour pH monitoring.

Usually, the evaluation begins with an EGD to exclude other causes of dysphagia such as a peptic stricture or a tumor. Typical findings are esophageal dilation and presence of retained food. Sometimes candidiasis of the esophageal mucosa is present. It is worth mentioning that gastroesophageal junction cancer infiltrating the LES may mimic achalasia. This misleading condition, called pseudo‐achalasia, should be ruled out in elderly patients, with short duration of symptoms and marked weight loss.^5^

The barium swallow often shows the characteristic “bird beak” sign (narrowing at the level of the gastroesophageal junction), delayed passage of the contrast into the stomach, an air‐fluid level, and tertiary contractions of the esophagus.

The gold standard for the diagnosis of achalasia is the high‐resolution esophageal manometry (HRM). It enables the measurement of the pressure, length, and relaxation of the lower and upper esophageal sphincters and assessment of esophageal peristalsis. To confirm the diagnosis of achalasia, it is necessary to document lack of esophageal peristalsis and partial or absent LES relaxation. The Chicago classification introduced by Pandolfino^6^ and his colleagues distinguishes three types of achalasia. Type I involves aperistalsis and absence of esophageal pressurization; type II is associated with aperistalsis and pan‐esophageal pressurization in at least 20% of swallows; and in type III there are premature spastic contractions (distal latency <4.5 seconds) in at least 20% of swallows. What makes the Chicago classification useful is that it can also help predicting treatment outcome. In fact, many studies have shown higher success rates in patients with type II achalasia.^7^, ^8^ It has been speculated that type II achalasia is an initial phase of the disease process with pan‐esophageal pressurization, while type I represents a later phase with complete absence of any contraction.^7^ Type III achalasia, characterized by premature spastic contractions, is associated with decreased response to surgical treatment.^7^ It is speculated that it may represent a recognizably different pathological process which is not a part of the progression from type II to type I achalasia, rather being a variant of distal esophageal spasm which involves the LES.^7^

Ambulatory pH monitoring is not necessary in the work‐up of patients with achalasia. It should be performed only in patients with heartburn and dysphagia who are considered to have gastroesophageal reflux refractory to medical treatment. In these patients, this test will distinguish GERD from achalasia.^9^ Interestingly, up to 50% of patients who end up having a diagnosis of achalasia have been treated for prolonged periods of time with proton pump inhibitors on the assumption that abnormal reflux was present.^10^ The pH monitoring study should also be performed after treatment to rule out pathologic gastroesophageal reflux (GER), which is often asymptomatic.^11^ In patients who are asymptomatic, particularly if young, we do prescribe acid‐reducing medications. In patients found to have erosive esophagitis, we also prescribe acid‐reducing medications.

## 3 SURGICAL TREATMENT OF ESOPHAGEAL ACHALASIA

### 3.1 Evolution of surgery for esophageal achalasia

In 1914, the first transabdominal extramucosal cardioplasty was described by Heller. He performed the myotomy both on the anterior and posterior walls of the cardia.^12^ Groeneveldt and Zaaijer simplified the procedure by performing one myotomy only.^13^ In the 1940s and 1950s, the transabdominal approach was the most commonly used, while few surgeons favored a left trans‐thoracic approach.^14^

Until the 1960s, the focus of treatment was on the relief of the dysphagia by the myotomy and no consideration was given to the possibility of post‐myotomy reflux. In 1956, Nissen popularized a 360‐degree fundoplication to control gastroesophageal reflux and this inspired Dor to propose a 180‐degree anterior fundoplication in 1962 that could be added to the myotomy.^15^ In 1963, Toupet described a partial posterior fundoplication.

At the beginning of the 1990s, minimally invasive techniques were introduced for the treatment of esophageal diseases. The first laparoscopic cardiomyotomy was performed by Cuschieri in 1991.^16^ In 1992, Pellegrini et al described the outcomes of myotomy performed through a left thoracoscopic approach, showing excellent results in about 90% of patients.^17^ However, it soon became evident that the procedure led to abnormal gastroesophageal reflux in 60% of patients.^18^ Their findings determined a switch to a laparoscopic approach combined with a partial fundoplication.^19^ In 1993, Ancona et al reported the technique of a laparoscopic esophageal myotomy and Dor fundoplication developed at the University of Padua.^20^ The same year the laparoscopic and open approach were compared showing that, while the outcomes were similar, the minimally invasive approach was associated with a shorter hospital stay, less post‐operative discomfort, and faster return to regular activities.^21^ Finally, at the end of 20th century, the laparoscopic Heller myotomy (LHM) with fundoplication became the standard of care worldwide (Table 1).

**Table 1:** Evolution of Minimally Invasive Surgery for Esophageal Achalasia

Year	Author	Importance
1991	Shimi et al^16^	Dr Cuschieri's group from the University of Dundee in United Kingdom performed the first laparoscopic Heller myotomy
1992	Pellegrini et al^17^	Dr Pellegrini from the University of California described the new technique of thoracoscopic Heller myotomy and performed the first minimally invasive cardiomyotomy in the USA
1993	Ancona et al^20^	The group from the University of Padua in Italy was first to report the technique of laparoscopic Heller myotomy with Dor fundoplication.
1995	Ancona et al^21^	Randomized trial comparing outcomes of laparoscopic and open Heller myotomy demonstrating the benefits of a minimally invasive approach
1998	Patti et al^18^	A comparison of thoracoscopic and laparoscopic Heller myotomy indicating high rate of postoperative reflux in patients after myotomy without fundoplication
1999	Patti et al^19^	Study showing long‐term outcomes of laparoscopic and thoracoscopic Heller myotomy indicating that laparoscopic Heller myotomy with Dor fundoplication should be considered the treatment of choice
2001	Melvin et al^22^	First case report of robotically assisted Heller myotomy
2004	Richards et al^23^	A randomized controlled study that confirmed the importance of adding an antireflux procedure to laparoscopic Heller myotomy in order to avoid postoperative reflux
2006	Torquati et al^24^	A report that confirmed good long‐term outcomes of laparoscopic Heller myotomy with Dor fundoplication in terms of symptom control and occurrence of postoperative reflux
2008	Rebecchi et al^25^	A randomized controlled trial that compared laparoscopic Heller myotomy with total and partial fundoplication and indicated higher rate of dysphagia symptoms after total fundoplication with no significant difference in postoperative reflux rate
2012	Rawlings et al^26^	A randomized study demonstrating the equivalence of anterior and posterior partial fundoplication after laparoscopic Heller myotomy in terms of symptom control and postoperative reflux
2019	Costantini^27^	A report of 25‐y experience at a single surgical center showing good long‐term outcomes of laparoscopic Heller myotomy with Dor fundoplication
2019	Werner et al^28^	First randomized controlled trial comparing outcomes of laparoscopic Heller myotomy with Dor fundoplication and peroral endoscopic myotomy demonstrating equivalence of both techniques in symptom control but higher rates of esophagitis after POEM

### 3.2 Laparoscopic Heller myotomy

The treatment of esophageal achalasia is palliative, and it focuses on decreasing the outflow resistance of the GEJ caused by the dysfunctional LES. LHM has been the gold standard therapy for most esophageal achalasia patients.^29^, ^30^ SAGES guidelines describe it as a safe and low‐risk treatment method for resolving symptoms and improving quality of life.^31^ This statement is based on strong evidence showing excellent and durable results.^27^, ^31^, ^32^, ^33^

The evolution of achalasia treatment clearly shows that a fundoplication is required to prevent postoperative GERD.^34^, ^35^ In 2003, Falkenback et al presented data from a prospective randomized trial in 20 open Heller myotomy patients comparing those with and without total fundoplication, at more than 3‐year follow‐up.^36^ By pH monitoring evaluation, they documented pathologic GER in 13.1% of patients with no fundoplication and 0.15% in the fundoplication group. In addition, Richards and colleagues,^23^ in a prospective and randomized double‐blind trial with 6‐month follow‐up, proved the superiority of LHM and anterior partial fundoplication versus LHM alone in terms of postoperative GER, by reporting 47.6% pathologic reflux after LHM alone, and only 9% after LHM with Dor fundoplication. Campos et al,^37^ in a large meta‐analysis, showed a higher rate of pathologic postoperative GER in patients after LHM alone versus LHM with fundoplication (32% vs 9%). These findings helped confirm that a fundoplication is necessary to control pathologic GER after myotomy.

Determining whether to perform a total or partial fundoplication was not clear from the start. Topart et al,^38^ in a 10‐year follow‐up evaluation of patients after LHM with total fundoplication, showed that 82% of the patients had recurrence of symptoms. In contrast, Rossetti et al^39^ described excellent outcomes regarding dysphagia symptoms relief in more than 90% of patients, showing no pathologic GER at mean follow‐up of 83 months. In 2008, Rebecchi and colleagues^25^ published data from their prospective randomized trial comparing the outcome of a LHM with a Dor or Nissen fundoplication. They found that at 5‐year follow‐up the postoperative pathologic GER ratio was similar in both groups. However, patients after total fundoplication had increased dysphagia rate when compared to those after Dor (15% vs 2.8%). Based on these findings, it is clear that a total fundoplication should not be performed in patients with achalasia after LHM, and LHM with partial fundoplication should be the treatment of choice.^27^

The best type of partial fundoplication (anterior or posterior) after LHM remains undetermined. A multicenter prospective trial by Rawlings et al^26^ indicated that at 1‐year follow‐up both procedures were equivalent in terms of symptom control and rates of pathologic GER. Kumagai and colleagues^40^ compared outcomes of LHM with Dor and Toupet fundoplication, finding no significant difference in postoperative pathologic GER and Eckardt score at 1‐year follow‐up. Since there is no evidence for the superiority of one type of partial fundoplication over the other, the choice should belong to the surgeon. Some prefer the partial anterior Dor fundoplication, which requires limited hiatal dissection and allows coverage of the exposed mucosa,^33^, ^41^, ^42^ while others believe that a partial posterior fundoplication may keep the edges of the myotomy separated, reducing the probability of recurrent dysphagia.^43^, ^44^

### 3.3 Technical aspects of LHM

Our technique for a laparoscopic Heller myotomy has been previously described in the literature.^45^ It consists of a 8 cm myotomy extending for 2.5 cm onto the gastric wall and a Dor fundoplication.

### 3.4 LHM vs other treatment options

Medical therapy and endoscopic botulin injection have limited effect and are indicated for patients who are not fit for other treatment modalities.^46^ Other options commonly used are pneumatic dilatation (PD) and the peroral endoscopic myotomy (POEM) (Table 2).

**Table 2:** Studies Comparing Different Treatment Modalities for Esophageal Achalasia

Source	Year	Design	Procedures	Group size [n]	F	Complication rate [%]	LOS [d]	Remission rate	Postoperative GERD
Ancona^21^	1995	RC	LHMD vs OHMD	34 (17 + 17)	6	0% (LHMD) vs 0% (OHMD)	4 (LHMD) vs 10 (OHMD)	94.2%(LHMD) vs 100%(OHMD)	by pH: 0% (THM) vs 5.8% (OHMD)
Patti^19^	1999	RC	THM vs LHMD/T	168 (35 + 133)	28	8.6% (THM) vs 5.2% (LHMD/T)	3 (THM) vs 2 (LHMD/T)	85% (THM) vs 93% (LHMD/T)	by pH: 60% (THM) vs 17% (LHMD/T)
Richards ^23^	2004	RCT	LHM vs LHMD	43 (21 + 22)	6	0% (LHM) vs 0% (LHMD)	1 (LHM) vs 1 (LHMD)	LHM = LHMD (*P* = .79)	by pH: 47.6% (LHM) vs 9.1% (LHMD) (*P* = .005)
Horgan^48^	2005	RC	RAHM vs LHMD	121 (59 + 62)	18	0% (RAHM) vs 16% (LHMD)	1.5 (RAHM) vs 2.2 (LHMD)	92% (RAHM) vs 90% (LHMD) (*P* = .5)	symptoms: 17% (RAHM) vs 16% (LHMD) (*P* = .9)
Mikaeli^49^	2006	RCT	PD vs EBTI + PD	54 (27 + 27)	12	0% (PD) vs 0% (EBTI + PD)	NA	62% (PD) vs 77% (EBTI + PD) (*P* = .1)	NA
Kostic^50^	2007	RCT	PD vs LHMT	51 (26 + 25)	12	8% (PD) vs 0% (LHMT)	0 (PD) vs 3 (LHMT)	77% (PD) vs 96% (LHMT) (*P* = .047)	NA
Rebecchi^25^	2008	RCT	LHMD vs LHMN	144 (72 + 72)	60	97% (LHMD) vs 85% (LHMN)	3.2 (LHMD) vs 3.6 (LHMN)	LHMD > LHMN (*P* = .001)	symptoms: 5.6% (LHMD) vs 0% (LHMN) by pH: 2.8% (LHMD) vs 0% (LHMN) (*P *> .05)
Bakhshipour^51^	2009	RCT	EBTI + PD vs PD	34 (16 + 18)	12	0% (EBTI + PD) vs 0% (PD)	NA	87.5% (EBTI + PD) vs. 55.5% (PD) (*P* = .53)	NA
Novais^52^	2010	RCT	PD vs LHMD	94 (4 + 47)	3	4% (PD) vs 0% (LHMD)	NA	73.8% (PD) vs 88.3% (LHMD) (*P* = .08)	by pH: 31% (PD) vs 4.7% (LHMD) (*P* = .001)
Boeckxstaens^53^	2011	RCT	PD vs LHMD	201 (95 + 106)	24	4% (PD) vs 12% (LHMD)	NA	86% (PD) vs 90% (LHMD) (*P* = .46)	NA
Rawlings^26^	2012	RCT	LHMD vs LHMT	60 (36 + 24)	12	5.6% (LHMD) vs 8.3% (LHMT)	NA	LHMD = LHMT (*P *> .05)	by pH: 41.7% (LHMD) vs 21.1% (LHMT) (*P* = .152)
Shaligram^54^	2012	RC	RAHM vs LHM vs OHM	2683 (149 + 2116 + 418)	1	4.02% (RAHM) vs 5.19% (LHM) vs 9.08% (Open‐HM)	2.42 (RAHM) vs 2.70 (LHM) vs 4.42 (OHM)	NA	NA
Borges^55^	2014	RCT	PD vs LHMD	92 (48 + 44)	24	4% (PD) vs 0% (LHMD)	NA	54% (PD) vs 60% (LHMD) (*P* = NS)	by pH: 27.7% (PD) vs 4.7% (LHMD) (*P* = .003)
Hamdy^56^	2015	RCT	PD vs LHMD	50 (25 + 25)	12	8% (PD) vs 4% (LHMD)	0 (PD) vs 3 (LHMD)	76% (PD) vs 96%(LHMD) (*P *= .04)	symptoms: 28% (PD) vs 16% (LHMD) (*P* = .3)
Persson^57^	2015	RCT	PD vs LHMT	53 (28 + 25)	60	0% (PD) vs 7% (LHMT)	NA	64% (PD) vs 92% (LHMT) (*P* = .016)	NA
Moonen^58^	2016	RCT	PD vs LHMD	201 (96 + 105)	60	5% (PD) vs 11% (LHMD)	NA	82% (PD) vs 84% (LHMD) (*P* = .92)	by pH: 12% (PD) vs 34% (LHMD) (*P* = .14)
Chrystoja^59^	2016	RCT	PD vs LHMD/T	50 (25 + 25)	60	4.5% (PD) vs 13% (LHMD/T)	NA	77% (PD) vs 100% (LHMD/T)	by pH: 10% (PD) vs 0% (LHMD/T) (*P* = .49)
Torres‐Villalobos^60^	2018	RCT	LHMD vs LHMT	73 (38 + 35)	24	2.6% (LHMD) vs 0% (LHMT)	2.54 (LHMD) vs 2.54 (LHMT)	100% (LHMD) vs 90% (LHMT)	by pH: 10.5% (LHMD vs 31.5% (LHMT) (*P* = .111)
Schlottmann^61^	2018	M	LHM vs POEM	7792 (5834 + 1958)	24	NA	POEM (+1.03 d) >LHMD	92.7% (POEM) vs 90% (LHM) (*P* = .01)	by pH: 11.1% (LHM) vs 47.5% (POEM) (*P* < .0001) EGD: 11.5% (LHM) vs 22.4% (POEM) (*P* < .0001)
Ponds^62^	2019	RCT	POEM vs PD	133 (67 + 66)	24	0% (POEM) vs 2% (PD)	NA	92% (POEM) vs 54% (PD) (*P* < .001)	by EGD: 41% (POEM) vs 7% (PD) (*P* = .002)
Costantini^63^	2019	CCS	POEM vs LHMD	240 (140 + 140)	24	5% (POEM) vs 2.1% (LHMD)	2 (POEM) vs 3 (LHMD)	99.3% (POEM) vs 97.1% (LHMD) (*P* < .12)	by pH: 38.4% (POEM) vs 17.1% (LHMD) (*P* < .01) by EGD: 37.4% (POEM) 15.2% (LHMD) (*P* < .05)
Werner^28^	2019	RCT	POEM vs LHMD	221 (109 + 112)	24	2.7% (POEM) vs 7.3% (LHMD)	POEM = LHMD (95% CI, −0.12‐0.63)	83% (POEM) vs 81.7% (LHMD) (*P* = .007 for noninferiority)	by pH: 30% (POEM) vs 30% (LHMD) by EGD: 44% (POEM) and 29% (LHMD) (95% CI 1.03‐3.85)

Abbreviations: CCS, case control study; EBTI, endoscopic botulin toxin injection; EGD, esophagogastroduodenoscopy; F, months of follow‐up; LHM, laparoscopic Heller myotomy; LHMD, laparoscopic Heller myotomy with Dor fundoplication; LHMD/T, laparoscopic Heller myotomy with Dor or Toupet fundoplication; LHMN, laparoscopic Heller myotomy with Nissen fundoplication; LHMT, laparoscopic Heller myotomy with Toupet fundoplication; LOS, length of stay; M, meta‐analysis; NA, data nonavailable; OHM, open Heller myotomy; OHMD, open Heller myotomy with Dor fundoplication; PD, Pneumatic dilation; pH, pH‐monitoring; POEM, peroral endoscopic myotomy; RAHM, robotically assisted Heller myotomy; RC, retrospective cohort; RCT, randomized controlled trial; THM, thoracoscopic Heller myotomy.

In 2015, a large European randomized controlled trial comparing LHM and PD was published.^58^ It showed no significant difference in success rate between the two treatments, with 84% and 82% success after 5 years for LHM and PD, respectively. However, 25% of patients treated with PD required additional dilatations. It is in fact known that patients treated with PD eventually require additional dilatations over time to control the symptoms. This was well shown in this randomized trial. In 2017, Ehlers et al^64^ also showed that LHM was associated with a lower rate of reintervention and readmission.

In 2010, Dr Inoue from Japan described a novel endoscopic technique – POEM.^47^ The myotomy was performed endoscopically by the creation of a long submucosal tunnel (mean length about 12 cm), followed by transection of the circular fibers for about 8 cm‐6 cm on the esophagus and 2 cm onto the gastric wall. Many retrospective studies from the United States, Asia, and Europe confirmed the initial experience, showing excellent relief of symptoms but a very high rate of post POEM pathologic reflux.^65^, ^66^, ^67^ Schlottmann et al,^61^ in a meta‐analysis of 54 studies, compared 5834 patients who underwent a LHM with 1958 patients treated with POEM, with an average follow‐up of 24 months. Their study indicated that POEM was slightly more effective than LHM, since the improvement rate of dysphagia was described in 92.7% of patients after POEM, and 90.0% of patients from LHM group. However, a significant difference was found in terms of pathologic GER. Ambulatory pH monitoring showed pathologic reflux in 48% of patients after POEM, but in only 11% of patients after LHM. Esophagitis was present in 22% of patients after POEM and in 12% after LHM. Kumbhari et al reported a higher rate of clinical response to POEM in patients with type III achalasia when compared to LHM with partial fundoplication (98.0% vs 80.8%).^68^ The reason for these different outcomes is probably due to the fact that POEM allows a proximally extended myotomy.

At the end of 2019, the results of a prospective European multicenter randomized trial comparing 109 patients who underwent LHM with 112 patients after POEM were published.^28^ At a 3‐month follow‐up, the rate of reflux esophagitis was 20% after LHM but 57% after POEM. The study indicated the equivalence of the two procedures in terms of symptom relief at 2‐year follow‐up, which was not surprising as POEM allows an excellent division of the muscle fibers. Overall, GER remains a major concern for POEM, particularly since there are data showing the onset of denovo Barrett's esophagus and reflux stricture after treatment.^69^ In addition, in 2019 the first case of esophageal cancer following POEM was reported.^70^

In patients with end stage of achalasia, many experts recommend an esophagectomy as primary treatment.^71^, ^72^ However, esophagectomy is associated with longer hospitalization, risk of pneumonia, anastomotic leak, recurrent laryngeal nerve injury, bleeding, chylothorax, and death.^72^, ^73^ Considering the satisfactory results of a myotomy, and the high morbidity and mortality associated with an esophagectomy, LHM should always be considered as the first‐line treatment option even in end‐stage achalasia, reserving esophagectomy for patients who have failed other treatment options.

## 4 FOLLOW‐UP

Achalasia patients have an increased risk of squamous cell cancer after treatment, usually 10 to 50 times higher than the general population.^74^, ^75^, ^76^ In addition, some studies have shown that adenocarcinoma can occur after treatment due to pathologic gastroesophageal reflux^77^, ^78^. Interestingly the group that designed the 2018 ISDE achalasia guidelines specifically said: “We make no recommendation about routine endoscopy surveillance or endoscopy intervals after any treatment”.^79^ In our center, we do recommend routine EGD every 3 years or when symptoms recur. Unfortunately, there are no precise guidelines regarding the timing and frequency of follow‐up EGD after intervention for achalasia. Even the 3‐year time frame is an arbitrary number that most but not all the insurance companies accept. Some insurance companies will allow an EGD only if a patient has recurrent symptoms.

## 5 TREATMENT ALGORITHM FOR ACHALASIA MANAGEMENT

POEM and LHM are equally effective and should considered in every patient with achalasia. In our center, we do perform LHM for patients with type I and type II achalasia. These patients are often overweight and have a hiatal hernia so that the addition of a fundoplication allows control of reflux in most patients. In patients with type III achalasia, POEM should be considered as initial treatment. In case of failure, we recommend PD as the second step therapy. If pneumatic dilatation fails, it is reasonable to consider POEM for those who underwent LHM initially and LHM for those after POEM. Esophagectomy should be considered as a last resort for patients with persisting symptoms after failure of other treatment modalities.

## DISCLOSURE

Conflict of Interests: Authors declare no conflict of interests for this article.
